# The Effect of Using Different Probiotics on the Performance, Digestibility of Nutrients, Feces Score, Blood Parameters and Skeletal Indices of Suckling Holstein's Calves

**DOI:** 10.1002/vms3.71136

**Published:** 2026-08-07

**Authors:** Mostafa Hosseinabadi, Kamel Amozadeh Araee, Taghi Ghoorchi, Ghasem Khadem, Ehsan Mirzadeh, Hamidreza Dosti

**Affiliations:** ^1^ Department of Animal and Poultry Nutrition Faculty of Animal Science Gorgan University of Agricultural Science and Natural Resources Gorgan Iran; ^2^ Vivan Co. Mashhad Iran

**Keywords:** faeces score, performance, probiotic, skeletal index, suckling calf

## Abstract

**Background:**

Rearing suckling calves is critical for dairy herd productivity, but this period often involves digestive disorders, poor nutrient digestibility and loose faeces, which impair growth. Probiotics are promising, yet their comprehensive effects on blood parameters and skeletal development in suckling Holstein calves remain unclear.

**Objectives:**

This study aimed to evaluate the effects of single and multi‐strain probiotics (*Saccharomyces boulardii*, *Bacillus subtilis* and *Lactobacillus*) on growth performance, faecal score, nutrient digestibility, blood metabolites and skeletal development in suckling Holstein calves.

**Methods:**

In this study, 36 Holstein female calves aged 7 ± 3 days with an initial body weight of 44.7 ± 2 kg were assigned to six treatments with six replicates in a completely randomized design for 45 days. Experimental treatments were as follows: (1) Control diet without probiotics; (2) diet containing *S. boulardii* (BS); (3) diet containing *B. subtilis* (B); (4) diet containing *Lactobacillus* (L); (5) diet containing *S. boulardii* + *Lactobacillus*; and (6) diet containing *S. boulardii* + *Lactobacillus* + *B. subtilis*. Probiotics were mixed with milk and administered once daily at a rate of 5 g per calf.

**Results:**

Probiotic supplementation significantly affected 28‐day body weight, weight gain from Days 1–28, final body weight, and overall daily weight gain (*p* < 0.05). Body weight change, total dry matter intake, daily starter intake, feed conversion ratio and dry matter intake were also improved by probiotic supplementation (*p* < 0.05). Final body weight in the control group was lower than in all probiotic‐treated groups (*p* < 0.05). Probiotic supplementation significantly improved faecal consistency score, with the lowest score observed in calves receiving *B. subtilis* (*p* < 0.05). Digestibility of dry matter, organic matter, crude fat and neutral detergent fibre increased with probiotic supplementation (*p* < 0.05). Blood biochemical parameters were not significantly affected by probiotic treatments. Skeletal indices were improved in probiotic‐treated calves compared to the control group (*p* < 0.05).

**Conclusions:**

Probiotic supplementation improved growth performance, faecal score, nutrient digestibility and skeletal development of suckling calves. Among the evaluated treatments, *B. subtilis* showed the most consistent positive response under the conditions of this study, although combined probiotic treatments also improved several parameters compared to the control.

## Graphical abstract

Adding probiotics to calf milk significantly improved growth, resulting in higher final body weight and daily weight gain compared to the control group. Probiotics, particularly Bacillus subtilis, enhanced fecal consistency scores, nutrient digestibility, and skeletal development. Multi strain combinations also showed positive effects on several performance parameters.

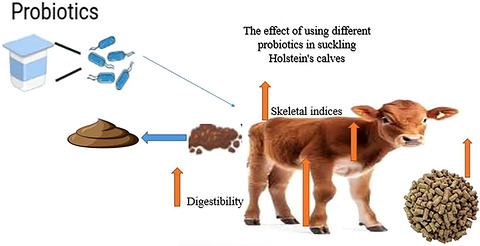

## 1 Introduction

Proper management during the first three months of life is critical for calf health, survival and future productive performance. In this critical period, the animal's physiological condition includes the ability to absorb macromolecules, especially immunoglobulin, from the intestine and the susceptibility to intestinal infection and diarrhoea (Ontsouka et al. 2016). Most importantly, the economic future of an industrial cattle farm is related to the breeding of replacement calves (Dobson et al. 2007). Most modern dairy farming systems require calves to be weaned immediately after birth and then artificially fed whole milk or milk replacer. As a result, new‐born calves cannot quickly acquire microflora from the mother's saliva and other cows. This slows the formation of microbial communities and can even create an unbalanced microbial flora in the digestive tract of calves (Lopes et al. 2021). Furthermore, if proper feeding and management strategies are not adopted during this critical life stage, it may negatively affect the growth rate and health status of calves and even production performance (Krpálková et al. 2014). Also, due to a series of specific characteristics in the body system, such as a weak defence system and a dynamic digestive system that is directed from the digestion of dairy products to solid materials, calves are more at risk of death than other animals (Heinrichs et al. 2003a).

Today, to improve livestock performance, the use of feed additives has increased. For many years, the use of antibiotics in calves has had many advantages, on the other hand, due to the antibiotic resistance of pathogens in calves whose milk contains antibiotics, their use in raising farm animals has become a sensitive issue. In addition, the harm that antibiotic resistance has for consumers and human health is increasing. Considering this issue, the use of probiotics and prebiotics has been proposed as the best alternative to the use of antibiotics in livestock (Alugongo, Xiao, Chung, et al. 2017; Hossein Abadi et al. 2021; Amozadeh Araee et al. 2023). Probiotics are defined as carefully selected live strains of microorganisms that, when administered in sufficient amounts, confer health benefits (such as reduced incidence of diarrhoea) to the host (Markowiak and Śliżewska 2017). The most common probiotics fed to young calves are live yeasts, mainly *Saccharomyces cerevisiae*, yeast cultures (Alugongo, Xiao, Wu, et al. 2017) and bacteria‐based probiotics such as *Lactobacillus*, *Bacillus* and *Enterococcus* (Uyeno et al. 2015).

*Saccharomyces boulardii* is a strain of *S. cerevisiae* and can reduce the risk of intestinal diseases by blocking the toxic site of pathogen destruction receptors, preventing the growth of some pathogens in the intestinal space, and modulating the immune system. Bacteria that become dominant in the intestine early on can affect the future growth of calves (He et al. 2017). *Bacillus subtilis* is a thermophilic, spore‐forming, Gram‐positive, motile bacterium with a rod‐shaped morphology (Mielich‐Süss and Lopez 2015). *B. subtilis* is a probiotic strain commonly used as a feed supplement for ruminants. It can enhance rumen fermentation and produce a diverse set of enzymes that facilitate intestinal absorption and digestion when consumed by livestock. In addition to facilitating the digestion of proteins and the return of urea to the rumen, *Bacillus* stimulates protein synthesis, facilitates the absorption of amino acids and peptides produced during digestion, and increases the number of proteins reaching the intestine (Wang et al. 2016). *B. subtilis* is capable of synthesizing polypeptides that are antagonistic to intestinal microorganisms, thereby significantly increasing the digestibility of animal feed. This aerobic bacterium maintains the ecological balance of the intestines by consuming oxygen and helping to create an anaerobic environment. This, in turn, promotes the reproduction of the dominant bacteria in the intestine. *Lactobacillus* are the dominant microbial population in the digestive tract of young milk‐fed animals, which defend the host by modulating the immune system (Fomenky et al. 2017).

The most important effects of supplementing yeast products during the pre‐weaning period have been reported when they are included in the diet of animals during stressful conditions, promoting rumen microbiota development and reducing the risk of pathogen colonization (Chaucheyras‐Durand and Durand 2010). Adding probiotics to the starter diet (Harris et al. 2017) or to milk (Brewer et al. 2014) improved feed intake, daily weight gain and faecal score compared with the control group. Also, the enrichment of milk with BS yeast improved DWG and improved performance compared to the control group (Galvão et al. 2005; Villot et al. 2019). The most common probiotic response is associated with a reduction in the incidence and severity of diarrhoea in livestock. Calves with failed passive immunity had fewer days of diarrhoea with the addition of *S. cerevisiae* (Galvão et al. 2005). In addition, calves fed yeast culture had better stool scores and overall health (Magalhães et al. 2008; Kim et al. 2011; Harris et al. 2017). Mortality rate and incidence of diarrhoea were reduced (Magalhães et al. 2008). Also, BS yeast supplementation in milk was associated with a lower incidence of severe diarrhoea and a tendency to reduce antibiotic treatments (Villot et al. 2019). It is known that these beneficial health effects lead to increased profitability, even without improved growth performance, due to reduced breeding costs (Magalhães et al. 2008). Probiotic supplementation can modulate gut microbial composition and enhance gut humoral immunity, effects that ultimately lead to reduced diarrhoea and improved growth performance (Abu‐Tarboush et al. 1996; Villot et al. 2019).

Although numerous studies have evaluated the effects of individual probiotic strains in calves, most research has focused on single‐strain supplementation, particularly *S. cerevisiae* or specific bacterial probiotics independently. Comparative studies assessing the simultaneous and combined effects of yeast‐ and bacteria‐based probiotics under identical management conditions remain limited, especially in suckling Holstein calves. Considering that different probiotic groups may act through distinct physiological and microbial mechanisms, combined supplementation could potentially result in complementary or synergistic effects on growth performance, nutrient utilization, gut health and skeletal development. Therefore, the present study was designed not only to evaluate individual probiotic strains but also to compare single and combined probiotic strategies in order to provide a clearer understanding of their relative and interactive effects in suckling calves.

## 2 Materials and Methods

### 2.1 Animals, Experimental Conditions and Experimental Diets

The present research was carried out in the breeding unit of 2500 Mehrmandgar dairy cows owned by Mr. Ahmad Qalandari located in Gorgan City, Iran. In this research, 36 suckling Holstein calves aged 7 ± 3 days and an initial body weight of 44.7 ± 2 kg were used in six treatments and six replications in the form of a completely randomized design for 45 days. Experimental treatments include 1‐ Experimental diet without probiotics (control), 2‐ Experimental diet containing *S. boulardii* yeast, 3‐ Experimental diet containing *B. subtilis*, 4‐ Experimental diet containing *Lactobacillus*, 5‐ Experimental diet containing *S. boulardii* yeast + *Lactobacillus* and 6‐ Experimental diet containing *S. boulardii* yeast + *Lactobacillus* + *B. subtilis*. Probiotics were mixed with milk and administered once daily at a rate of 5 g per calf, according to the manufacturer's recommendation.

In this research, the calves were kept in an individual place (1 × 1.20 m^2^) with a concrete floor and a bed of stubble that had already been flamed and disinfected. Calves were fed daily with two servings of whole milk at the rate of 15% of their body weight during infancy. All the calves were placed in the same management and nutritional conditions and the starter feed plus alfalfa (90:10 ratio) was provided to the calves and their daily intake was recorded. Every morning at 7 AM, the feed was given to the calves after weighing. Also, clean water was freely available to the calves during the experiment. The percentage of food and nutrients in the diets of the treatments is shown in Table 1.

**TABLE 1:** Constituent feed ingredients and chemical composition of the ration of suckling Holstein calves.

Feed components (%DM)	%	Chemical compounds	%
Alfalfa	10	Dry matter (%)	0.89
Barley seed	20	Crude protein (%)	21.75
Corn seed	44	Crude fat (%)	2.24
Wheat bran	5	Acid detergent fibre (%)	7.48
Soybean meal	18.51	Neutral detergent fibre (%)	21.35
Vitamin‐mineral supplement^a^	1.2	Ash (%)	3.89
Zista supplement^b^	0.09	Metabolizable energy (Mcal/kg)	2.86
Toxin binder	0.2	Non‐fibre carbohydrates^c^	50.77
Sodium bicarbonate	1		

^a^ Vitamin and mineral premix provided per kilogram of ration: Vitamin A, IU 1000000; Vitamin IU, D3 250000; Vitamin IU, E 3000; Magnesium, 32,000 mg; Manganese, 10,000 mg; Zinc, 10000 mg; Copper, 300 mg; Selenium, 100 mg; Calcium, 100 mg; Iron, 3000 mg; Cobalt, 100 mg; Phosphorus, 30,000 mg; Monensin, 1500 mg; Antioxidant, 100 mg.

^b^ Contains trace minerals cobalt, iodine, selenium, zinc, manganese, iron and copper.

^c^ Non‐fibre carbohydrates = 100 – (%Crude protein — %Neutral detergent fibre — %Crude fat — %Ash).

### 2.2 Preparation of Probiotics

The probiotics required for this research were obtained from Vivan Tak ecsir Iranian Company (Mashhad, Iran) and then transferred to the feed store and fed to the calves after mixing with milk.

Probiotics included the yeast *S. boulardii* and *B. subtilis*, and *Lactobacillus* included a number of different bacteria from the *Lactobacillus* family.

The Viable Colony‐forming Unit (CFU) Count

*S. Boulardii*: 3 × 10^9^ CFU/G

*B. subtilis*: 3 × 10^8^ CFU/G

*Lactobacillus*: 1.2 × 10^8^ CFU/G

### 2.3 Performance Measurement of Calves

The rations used in this experiment were provided to the calves every day at 7 AM and 4 PM. The feed given and the remaining feed for each animal were weighed and recorded every day. The daily feed intake was calculated from the average difference between the feed given to each animal and the remaining feed of the same animal the next day. The average of each treatment was calculated from the average feed consumed by each animal during the period. Also, the increase in the amount of feed given to the animals was determined based on the post‐feeding of each animal on the next day, so that if the animal left less than 200 gr of post‐feeding in 3 consecutive days, the feed of the animal would increase. The same procedure was carried out until the end of the test period. Calves were weighed every two weeks (0, 14, 28, 42 days of the experiment) fasting, after 16 h of starvation, using a digital scale. To calculate the DWG was calculated by dividing the weight difference in a period by the number of days in the same period. The amount of DMI, DWG, weight at the end of the measurement period and FCR were calculated. The amount FCR was also calculated by dividing the average amount of DMI by each calf at the end of the period by the DWG of the same animal in the entire period and as an average of the conversion factor of each treatment. Performance parameters were measured according to standard procedures described by (Amozadeh Araee et al. 2023; Khadem et al. 2025).

### 2.4 Measuring the Health Index of Calves

To check the health index of the calves, during the daily test, the appearance of faeces was evaluated. Scoring of faeces score was considered as an indicator of animal health daily for each of the calves. Faeces' score including 4 points from 1 to 4 was done as follows. A score of 1 for natural faeces (solid natural faeces that retain their physical shape after falling on the site), a score of 2 for soft faeces (heaps of faeces that spread after falling on the site and the solid part is more than liquid), a score of 3 was given for loose faeces (faeces that spread after falling onto the floor, with an equal amount of liquid and solid parts and flowing on the floor), and a score of 4 was considered for watery faeces (faeces that are watery and mucous) (Larson et al. 1977). If the average faeces score for stool fluidity and consistency is ≤ 3, 1 day of diarrhoea was recorded for that calf.

### 2.5 Measurement of Nutrient Digestibility

To measure the apparent digestibility of nutrients, the internal marker of acid‐insoluble ash (AIA) was used (Van Keulen and Young 1977). During the last three days of the experiment, the samples collected from each calf were mixed and a 100 gr stool sample was placed in a plastic nylon bag for chemical analysis, and the lid was closed and frozen at −20°C. Then, the percentage of dry matter (DM), organic matter (OM), ethereal extract (EE) and neutral detergent fibres (NDF) of the samples was determined in the laboratory based on the methods of (AOAC 2005) and (Van Soest, 1994).

### 2.6 Measurement of Blood Parameters

Blood sampling to measure blood parameters on the last day of the experiment (45 days) was taken from the jugular vein (10 mL) without using an anticoagulant and sent to the laboratory quickly in a flask containing ice. The tubes were centrifuged at 3000 × *g* for 10 min to separate the serum. The values ​​of glucose, triglyceride, cholesterol and blood urea nitrogen were measured by the commercial kits of the delta darman part using an autoanalyzer (Model BT 1500) made in Italy.

### 2.7 Measurement of Skeletal Indices of Calves

The measurement of some physical quantities such as the muzzle width, eyes interval, horns interval, chest, abdomen environment, height of rump, height of the seat and body length were measured every two weeks (14, 28, 42 days of the experiment). For this purpose, the calves were kept on a completely flat and clean surface, then the body dimensions were measured and recorded with a meter and caliper.

### 2.8 Statistical Model

The obtained data were statistically analysed in the form of a completely randomized design with six treatments and six repetitions using (SAS 2004) (9.1). Data related to performance, digestibility of nutrients, health index, blood parameters and skeletal indices (using GLM procedure) and weight data were analysed in different periods in a time‐repeated design based on the Mixed procedure. Also, the average treatments were compared through Duncan's test at a significance level of 5%. The model used for this design is as follows:

The model used for this design is as follows:

(1) yij=μ+Ti+εij

where as *yij* is the value of the *i*th observation in the *j*th repetition, *μ* is the average effect, *Ti* is effect of *i*th treatment, *εij* is the effect of experimental error related to the *i*th treatment in the *j*th repetition.

(2) yij=μ+Ti+Bj+(T×B)ij+εij

In this relation, *yij*, is the value of each observation, *μ* is the mean of all observations, *Ti* is the treatment effect, *Bj* is the time effect; (*T* × *B*)*ij* is the fixed effect of time and treatment and *εij* is the experimental error effect.

## 3 Results

### 3.1 Performance

**FIGURE 1:**
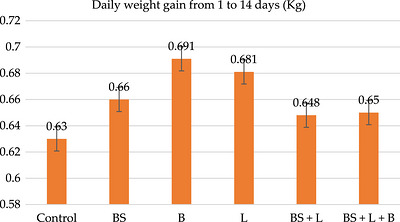
The effect of adding different probiotics on the weight gain of 1‐14‐day‐old suckling Holstein calves.

**FIGURE 2:**
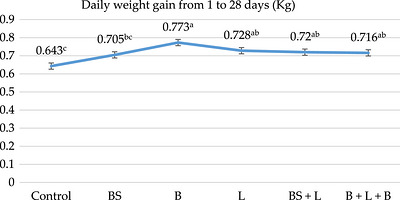
The effect of adding different probiotics on the weight gain of 1‐28‐day‐old suckling Holstein calves.

Information about the performance of suckling calves receiving different probiotics is given in Table 2, Table 3 and Figures 1 and  2. According to the results of the present study, probiotic consumption caused a significant difference in 28‐day weight, 1‐28‐day weight gain, final body weight and DWG of the entire period (*p* < 0.05). Also, when comparing the body weight of calves in the probiotic‐receiving treatments in terms of average calf weight and calf age, the control group was lower than the other treatments (*p* < 0.05). BWC, TDMI for the entire period, DSI, FCR and DMI were improved with the use of probiotics in calf milk (*p* < 0.05). B consumption caused a significant difference in 28‐day weight and DWG from 1 to 28 days with control and BS treatments (*p* < 0.05); while it did not make a significant difference with the other three treatments. On the other hand, the final weight of the control treatment was lower than other experimental treatments (*p* < 0.05). But the calves receiving BS yeast with L, BS + B and BS + L + B had no significant differences with each other. While the final weight of calves receiving B was higher than other treatments (*p* < 0.05). According to the results of the present study, more changes in BWC were observed with intake in calves (*p* < 0.05). The highest BWC were related to the treatment receiving B (32.23 kg), and the lowest were related to the control treatment (27.24 kg). No significant difference was observed between the calves receiving B and L, BS + B, and BS + L + B in terms of weight gain during the entire period, although a significant difference was observed between the control and BS treatment (*p* < 0.05). Among the experimental treatments, the calves receiving B had the lowest (1.24) and the control group had the highest (1.35) FCR (*p* < 0.05). While the FCR of calves receiving different probiotics was not significantly different. The control and BS treatments did not have significant differences with each other in DMI, while they had significant differences with other experimental treatments (*p* < 0.05). Also, the addition of B and L did not cause a significant difference in DWG. In addition, the use of a combination of different probiotics did not lead to significant differences with each other. According to the present results, consumption of different probiotics has no significant effect on 14‐day weight, DWG from 1 to 14 days and daily milk intake.

**TABLE 2:** Effect of adding different probiotics on the weight of Holstein suckling calves.

Trait	Experimental diets						Average age of calves
	Control	BS	B	L	BS + L	BS + L + B	
14th day weight (kg)	53.44^z^ ± 0.37	53.87^z^ ± 0.37	54.48^z^ ± 0.37	54.40^z^ ± 0.37	53.79^z^ ± 0.37	53.80^z^ ± 0.37	53.96^z^ ± 0.17
28th day weight (kg)	62.03^c,y^ ± 0.37	63.82^b,y^ ± 0.37	65.81^a,y^ ± 0.37	64.59^b,y^ ± 0.37	64.35^b,y^ ± 0.37	64.23^b,y^ ± 0.37	64.14^y^ ± 0.17
42th day weight (kg)	71.19 ^c,x^ ± 0.37	73.78^b,x^ ± 0.37	76.26^a,x^ ± 0.37	74.73^b,x^ ± 0.37	74.22^b,x^ ± 0.37	74.11^b,x^ ± 0.37	74.05^x^ ± 0.17
Average treatments	62.22^d^ ± 0.21	63.82^c^ ± 0.21	65.51^a^ ± 0.21	64.57^b^ ± 0.21	64.12^bc^ ± 0.21	64.05^bc^ ± 0.21	

*Note*: a, b, c: The presence of dissimilar letters in each column indicates a significant difference in different ages (*p* < 0.05). x, y, z: The presence of dissimilar letters in each row indicates a significant difference between different treatments (*p* < 0.05).

**TABLE 3:** The effect of adding different probiotics on the performance of suckling Holstein calves.

Trait	Experimental diets							
	Control	BS	B	L	BS + L	BS + L + B	SEM	*p*‐value
Initial weight (kg)	43.95	43.94	44.03	44.08	44.01	43.98	0.44	0.999
BWC (kg)	27.24^c^	29.84^b^	32.23^a^	30.65^ab^	30.21^ab^	30.13^ab^	0.488	< 0.001
DWG (kg/day)	0.643^c^	0.706^b^	0.761^a^	0.725^ab^	0.716^ab^	0.713^ab^	0.011	< 0.001
TDMI (kg)	36.83^d^	37.56^c^	40.27^a^	39.72^a^	38.68^b^	38.53^b^	0.156	< 0.001
DSI (kg/day)	0.870^d^	0.890^c^	0.953^a^	0.941^a^	0.918^b^	0.915^b^	0.004	< 0.001
FCR	1.35^a^	1.25^b^	1.24^b^	1.29^ab^	1.28^ab^	1.27^ab^	0.02	0.014
Daily milk intake (g)	739.79	742.08	746.04	743.25	741.25	740.2	3.828	0.864
DMI (g)	1616.8^c^	1636.4^c^	1704.9^a^	1689.3^a^	1662.4^b^	1660.2^b^	4.735	0.001

*Note*: Means in columns with different superscripts differ significantly (*p* < 0.05).

Abbreviation: SEM, standard error of means.

### 3.2 Nutrient Digestibility

Table 4 shows the measured data of nutrient digestibility changes of Holstein suckling calves during the experiment. The results of the present study show that the addition of different probiotics to calves' milk improves the digestibility of DM, OM, crude fat and NDF (*p* < 0.05). Among the treatments receiving B with L, BS + L and BS + L + B, as well as the control treatment with BS, no significant difference was observed in terms of digestibility of DM and crude fat. In terms of the digestibility of OM, no significant difference was observed among the calves receiving BS with L, BS + L and BS + L + BS. Meanwhile, there was a significant difference between the calves receiving BS and B and the control group in terms of digestibility of OM (*p* < 0.05). According to the results of the present study, there was crude fat digestibility between the control groups and BS with B, BS + L and BS + L + BS (*p* < 0.05). In terms of digestibility of insoluble fibres in neutral detergent, there was no significant difference between the BS, L and BS + L treatments with each other and the group receiving BS with BS + L and BS + L + B.

**TABLE 4:** Effect of adding different probiotics on nutrient digestibility of suckling Holstein calves (%).

Trait	Experimental diets						SEM	*p*‐value
	Control	BS	B	L	BS + L	BS + L + B		
DM	78.31^c^	82.26^b^	85.34^a^	85.11^a^	84.03^ab^	83.81^ab^	0.452	<0.001
OM	70.86^c^	74.74^b^	78.60^a^	76.96^ab^	75.27^ab^	75.02^b^	0.612	<0.001
EE	69.69^b^	70.06^b^	74.35^a^	74.01^a^	72.78^a^	72.68^a^	0.354	<0.001
NDF	57.78^d^	60.79^c^	64.70^a^	63.78^ab^	63.69^bac^	61.78^bc^	0.483	<0.001

*Note*: Means in columns with different superscripts differ significantly (*p* < 0.05).

Abbreviation: SEM, standard error of means.

### 3.3 Faeces Score

The results of faeces score in suckling calves are shown in Table 5. According to the results of the present study, the addition of different probiotics to the milk of suckling calves increased the faeces score (*p* < 0.05). Meanwhile, the control treatment had the highest (2.46) and the treatment receiving B had the lowest (1.82) stool score. According to Table 4, there was no significant difference between the control group and the calves receiving BS yeast, L, BS + L and BS + L + B, although the use of probiotics has improved faeces score numerically. Also, according to the results of the present study, no significant difference was observed in calves receiving different probiotics. Among the consumed probiotics, calves receiving B probiotics had better faeces scores.

**TABLE 5:** The effect of adding different probiotics on the health index of suckling Holstein calves.

	Experimental diets							
Trait	Control	BS	B	L	BS + L	BS + L + B	SEM	*p*‐value
Faeces score	2.46^a^	2.01^ab^	1.82^b^	1.92^ab^	1.96^ab^	1.93^ab^	0.138	0.038

*Note*: Means in columns with different superscripts differ significantly (*p* < 0.05).

Abbreviation: SEM, standard error of means.

### 3.4 Blood Parameters

The information obtained from the results of the effect of different probiotics on blood parameters is presented in Table 6. According to the results of our research, the addition of different probiotics to the milk of suckling calves did not have a significant effect on the concentration of cholesterol, triglycerides, glucose, urea nitrogen, total protein, albumin, globulin and the ratio of these two. In terms of non‐significance, the concentration of glucose, total protein, albumin, globulin and the ratio of albumin to globulin of calves increased by adding to milk, while the concentration of cholesterol, triglyceride and urea nitrogen had a decreasing trend, which was not statistically significant. According to the results, the highest (92.50 mg/dL) and the lowest (85.75 mg/dL) glucose concentration were related to the calves receiving B and the control.

**TABLE 6:** The effect of adding different probiotics on the blood parameters of suckling Holstein calves.

Trait	Experimental diets							
	Control	BS	B	L	BS + L	BS + L + B	SEM	*p*‐value
Cholesterol (mg/dL)	93.75	93.25	92.25	91.75	92	92.75	0.961	0.822
Triglyceride (mg/dL)	35	32.75	34	34.5	32.75	32.5	3.718	0.997
Glucose (mg/dL)	85.75	89	92.5	88.25	87.25	87.75	1.721	0.362
Urea (mg/dL)	18	16.5	14.5	16.25	16.5	16.55	1.003	0.549
Total protein (g/dL)	5.96	6.23	6.34	6.31	6.33	6.28	0.109	0.358
Albumin (g/dL)	3.49	3.57	3.81	3.53	3.73	3.63	0.077	0.188
Globulin (g/dL)	2.12	2.16	2.17	2.14	2.15	2.17	0.042	0.975
Albumin/Globulin	1.64	1.65	1.75	1.64	1.73	1.67	0.053	0.738

*Note*: Means in columns with different superscripts differ significantly (*p* < 0.05).

Abbreviation: SEM, standard error of means.

### 3.5 Skeletal Index

The information related to the effect of using different probiotics on the skeletal index of calves every two weeks is given in Table 7. According to the results of the present study, adding probiotics to the milk of infant calves did not have a significant effect on the distance muzzle width, eyes interval and horns interval in the entire experimental period, the size of the abdominal environment at two weeks old and the height from the withers at six weeks old. However, the size of the chest and height of the rump of the two, four and six‐week‐old calves receiving B was higher than the other experimental treatments, which had a significant difference with the control group (*p* < 0.05), although no significant difference was observed between the receiving treatments. The height of calves receiving different probiotics was higher than the control group (*p* < 0.05), although no significant difference was observed between the treatment receiving BS yeast and the control group. While there was no significant difference in the height of the seat at four weeks of age among the calves receiving different probiotics, there was a significant difference between the calves receiving B and the control group (*p* < 0.05). According to the results of the present study, the calves receiving different probiotics had more body length at two weeks than the control group (*p* < 0.05), but the body length of the control group calves was not significantly different from BS. According to the size of the abdominal environment in four‐week‐old calves, consumption of B has caused a significant increase in this parameter compared to the control group (*p* < 0.05). However, the treatments receiving BS yeast, L, BS + L and BS + L + B were not significantly different from the control group. In the six‐week measurement, the abdominal environment of the calves receiving different probiotics was higher than the control group (*p* < 0.05). However, no significant difference was observed between the treatments receiving probiotics with each other and the control calves receiving BS + L. The results of the present study show that the addition of B to the milk of the infant calves improved the height of the head of the calves at four and six weeks of age and the body length of the calves at four weeks of age compared to the control group (*p* < 0.05). This was even though there was no significant difference with other experimental treatments. Also, there was no significant difference between the calves of the control group and the calves receiving BS yeast and BS + L in terms of body length at four and six weeks.

**TABLE 7:** The effect of adding different probiotics on the skeletal index of Holstein calves (cm).

Trait	Experimental diets						SEM	*p*‐value
	Control	BS	B	L	BS + L	BS + L + B		
**Skeletal indices in the two‐week experiment**								
Muzzle width	7	7.25	7.38	7.36	7.36	7.21	0.362	0.937
Eyes interval	13.5	13.66	13.71	13.71	13.83	13.7	0.361	0.99
Horns interval	10.66	10.7	10.76	10.65	10.78	10.73	0.198	0.995
Chest	81.16^b^	84.50^a^	85.16^a^	83.83^ab^	84.00^a^	84.83^a^	0.748	0.006
Abdominal environment	82.33	83.33	84.66	84.33	83.66	84.16	0.828	0.416
Height of rump	81.66^b^	83.66^ab^	85.50^a^	84.00^a^	83.83^ab^	83.83^ab^	0.526	< 0.001
Height of the seat	78.16^c^	80.33^bc^	84.16^a^	83.50^a^	81.66^ab^	82.23^ab^	0.704	< 0.001
Body length	89.33^d^	90.83^dc^	94.83^a^	94.00^ab^	92.00^ab^	93.33^abc^	0.588	< 0.001
**Skeletal indices in the four‐week experiment**								
Muzzle width	7	7.25	7.38	7.36	7.36	7.21	0.362	0.937
Eyes interval	15.71	15.76	15.88	15.85	15.7	15.96	0.405	0.996
Horns interval	12.63	12.68	12.88	12.85	12.73	12.83	0.174	0.887
Chest	101.66^b^	105.16^ab^	107.00^a^	104.00^ab^	105.83^ab^	105.16^ab^	0.11	0.039
Abdominal environment	103.66^b^	104.83^ab^	106.83^ab^	105.16^ab^	104.66^ab^	105.50^ab^	0.686	0.066
Height of rump	90.83^b^	93.00^ab^	95.00^a^	94.33^a^	93.50^a^	93.83^a^	0.598	0.007
Height of the seat	87.33^b^	90.16^ab^	93.83^a^	92.83^ab^	90.66^ab^	91.33^ab^	1.411	0.046
Body length	94.33^c^	95.83^bc^	98.33^a^	98.16^ab^	96.66^abc^	97.77^ab^	0.543	0.001
**Skeletal indices in the six‐week experiment**								
Muzzle width	8.05	8.2	8.38	8.33	8.31	8.23	0.36	0.99
Eyes interval	15.71	15.76	15.88	15.85	15.7	15.96	0.405	0.996
Horns interval	14.56	14.7	14.96	14.75	14.88	14.86	0.146	0.439
Chest	119.66^b^	122.33^ab^	125.33^a^	123.33^a^	124.00^a^	123.83^a^	0.723	0.002
Abdominal environment	121.33^b^	124.33^a^	126.33^a^	125.00^a^	123.83^ab^	125.66^a^	0.648	< 0.001
Height of rump	100.16^c^	103.66^b^	106.16^a^	104.83^ab^	103.50^b^	104.66^ab^	0.58	0.001
Height of the seat	101.5	101.16	104	102.83	101.66	101.33	0.823	0.139
Body length	99.16^c^	101.00^bc^	104.00^a^	103.00^ab^	101.66^bac^	103.50^ab^	0.604	0.001

*Note*: Means in columns with different superscripts differ significantly (*p* < 0.05).

Abbreviations: SEM, standard error of means.

## 4 Discussion

Globally, the use of probiotics as an alternative to antibiotics in agriculture has increased due to increasing concerns about antibiotic resistance (Hume 2011). A variety of microbial species such as B, *Enterococcus* and *S. cerevisiae* (Simon et al. 2001). It has been used in livestock as probiotic products. The use of microbial additives leads to an increase in DMI (Desnoyers et al. 2009; Yousif et al. 2018; Hassan et al. 2020) and it has been found that increasing feed intake affects livestock performance. In agreement with the current research, Ahmed et al. (2022) reported that adding B to the milk of lactating calves improves body weight, increased weight, DMI and FCR for one month, and also stated that calves receiving 10 and 20 gr of B performed better were 60 days old. It should be noted that Ahmed et al. (2022) used higher probiotic inclusion levels (10–20 g per calf per day) compared with the present study (5 g per calf per day). Despite the lower inclusion rate, significant improvements were observed in growth performance in the current experiment. This difference may be related to variations in probiotic strains, microbial concentration, formulation and viability. Therefore, probiotic efficacy depends not only on the inclusion level but also on strain characteristics and product quality. This result is consistent with Smock et al. (2020) who reported improvement in final weight and DWG of calves receiving B. Also, they found that buffalo calves (Mousa and Marwan 2019), goats (Khalifa et al. 2016) and Holstein suckling calves receiving B had higher body weight than the control group (Kowalski et al. 2009). In line with our research, Mehrdad et al. (2017) reported that adding *S. cerevisiae* yeast to the ratio of suckling calves improved their growth performance. The use of *S. cerevisiae* and BS yeast has improved the final weight of suckling calves (Galvão et al. 2005). Also, Payandeh and Kafilzadeh (2007) stated that supplementing lambs' diet with yeast increased their growth performance, but had no effect on FCR. Hossein Abadi et al. (2018) stated in research that BWC, DWG and feed efficiency were not significantly different between calves receiving yeast, although the average DMI between the control group and the group that consumed yeast was significant. The study of Didarkhah and Bashtani (2018) shows that supplementing probiotics in the diet has a significant effect on performance parameters such as average DMI, FCR, final weight and DWG. Calves supplemented with *S. cerevisiae* showed higher DWG over 15–21 days (Harris et al. 2015). Most of the researchers' results show that adding yeast to livestock diets improves feed intake (Williams et al. 1991); DWG, rumen fermentation and the population of microorganisms in the rumen, especially cellulose‐digesting bacteria (Dawson et al. 1990) and also play a role in the absorption of nutrients and their metabolism (Williams et al. 1991). A study conducted on 42 male Holstein calves showed that the consumption of BS yeast did not affect their DMI, but the DMI increased with age (He et al. 2017). The study of Fokkink et al. (2009) shows that adding two percent of yeast DM to Holstein calf rations increases DWG. Smock et al. (2020) adding *B. subtilis* to the ration of dairy cows has improved DWG and final weight. Also, Liao et al. (2010) and Kowalski et al. (2009) showed that the supplementation of bacterial probiotics improves DMI and FCR in the lactation and postpartum period of cows compared to the control. In another study, which was conducted on 120 male Holstein calves in two control and treatment groups receiving 1.5 gr of *S. cerevisiae* yeast, it was shown that the DWG at the age of 35 days was significantly higher than the control group and also their performance in after weaning, it was better than the control group (Terré et al. 2015).

The improvement in the performance of livestock‐consuming yeast can be attributed to the better growth of the rumen, which will increase the absorption of nutrients, especially butyrate produced by *Butyrivibrio* bacteria from the rumen (Xiao et al. 2016). Also, probiotics in the digestive tract reduce the activity of harmful bacteria, which improves the absorption of nutrients reduces the conditions for the release of undigested nutrients in the rumen, and causes more weight gain (Hutjens 1996). The results of several researches on the effect of using yeast as a bio‐adjuvant in the feed of animals showed that by consuming oxygen in the rumen, yeast provides favourable anaerobic conditions for the activity of anaerobic microbes, which cause the improvement and growth of these groups of microorganisms (Dann et al. 2000). Also, another reason for improved performance can be pointed to the better growth of the rumen and the increase in the length and width of the villi, which increases the absorption of nutrients in the rumen (Alugongo, Xiao, Chung, et al. 2017). *Bacillus* can produce metabolites such as short‐chain fatty acids and enzymes that help increase DM intake, improve digestion and nutrient absorption. These metabolites can also help strengthen the calves' immune system (Chong 2009; Ahmed et al. 2022). The superior response observed in calves receiving *B. subtilis* may be related to the specific characteristics of this microorganism. *Bacillus* species are spore‐forming bacteria with high resistance to environmental and gastrointestinal stresses, which improves their survival rate in the digestive tract of young calves. In addition, *B. subtilis* is known to produce a wide range of extracellular enzymes, including proteases, amylases and lipases, which can enhance nutrient digestibility and feed utilization. The lack of a consistent synergistic response in the combined treatments may be associated with strain compatibility, dosage level or the relatively short duration of the experiment. Therefore, the better performance observed in the *B. subtilis* group may be attributed to its greater stability and direct effects on digestive efficiency and gut health (Wang et al. 2016; Fomenky et al. 2017).

Contrary to the results of the present study, the results show that the addition of yeast to the diet of suckling calves and dairy cows caused a significant decrease in feed intake (Saremi et al. 2003). Hossein Abadi et al. (2018) reported that enriching milk or starter feed of calves with bacterial prebiotics has no significant difference in final body weight and DWG. Galvão et al. (2005) showed that adding *S. cerevisiae* to the starter diet increased feed intake and DWG in calves, but no significant effect was observed when yeast was added to milk. Also, Chong (2009) reported that the use of yeast does not affect DWG. When 1 g of BS yeast was added to milk, it did not affect the DMI and performance of young calves (Pinos‐Rodríguez et al. 2008). Titi et al. (2008) reported that yeast supplementation did not affect DWG in lambs or goats. In addition, several researchers reported that feed efficiency was not affected by the yeast *S. cerevisiae* (Lesmeister et al. 2004; Magalhães et al. 2008; Alugongo, Xiao, Wu, et al. 2017). Because yeast could not significantly increase feed intake and DWG in these studies, subsequently it did not have positive effects on feed efficiency. Different responses to feeding probiotic products in intake and DWG may be related to the type of yeast product (live yeast vs. cultured yeast), probiotic strain, amount fed to livestock and method of yeast administration (in milk vs. starter feed).

DMI is influenced by insoluble fibres in a neutral detergent, the kinetics of rumen digestion, and the nature of feed decomposition in the rumen. Insoluble fibre in neutral detergent has a negative correlation of 0.65 with DMI (Beauchemin and Yang 2005), which is the first component to limit feed intake in the diet due to slow digestion and bulkiness (Ben Salem et al. 2008). The presence of physical receptors in the rumen and their sensitivity to pressure and even chemical metabolites control feed intake. Chemical receptors are sensitive to volatile fatty acids and pH and in this way affect feed intake (Beauchemin and Yang 2005). Haddad and Goussous (2005) stated that supplementing probiotics to calf diets improved the digestibility of insoluble fibres in acidic detergent. Also, numerous studies show the improvement of crude protein digestibility, OM and the number of proteolytic bacteria in the rumen of calves receiving *S. cerevisiae* yeast (Plata et al. 1994; Yoon and Stern 1996; Cavini et al. 2015). The results of Didarkhah and Bashtani (2018) showed that the intake of probiotics improved the apparent digestibility of DM and OM of nutrients, although it did not affect the digestibility of protein and crude fat.

In most of the studies and research carried out on the effect of yeast on the digestibility of nutrients, a process of improvement and numerical increase was observed (Erasmus et al. 1992; Cavani et al. 2015). However, this increase was sometimes not enough to observe a significant difference between the treatments. This reason can be due to the type of experimental animal (Titi et al. 2008). The consumed ration, the amount of intake, the strain and duration of yeast consumption and the physiological state of the consuming animal (Robinson 1997). In another study, some researchers reported that the addition of probiotics to the diet of suckling calves did not affect the ability to digest nutrients, including DM, OM and NDF (Hossein Abadi et al. 2018). Improving the digestibility of nutrients in animals consuming yeast can be due to the activation of the microbial population, which is influenced by the ability of yeast to remove oxygen from the rumen fluid and improve the anaerobic environment of the rumen (Plata et al. 1994). And *Bacillus* can produce metabolites such as short‐chain fatty acids, vitamins and enzymes that help improve digestion and nutrient absorption. These metabolites can also help lower the pH of the gut and prevent the growth of harmful bacteria (Ahmed et al. 2022).

A calf that is in good health will have a high growth rate and as a result, will perform better in the future. In the first months of their life, calves are very sensitive to diseases caused by various pathogens and environmental stressors, because their immune system is not yet fully active. In general, consuming a sufficient amount of high‐quality colostrum in the first 24 h of calf life is important to obtain passive immunity (Maamouri and Ben Salem 2021), which has an inverse relationship with the mortality rate and complications of calves (He et al. 2017). The growth and development of the microbial population of the rumen, and intestine and the as soon as possible acclimatization of the lactating animals to dry feed are the two main goals in suppressing pathogenic agents causing diarrhoea (He et al. 2017). Therefore, it is expected that the consumption of probiotics with microbial balance in the digestive system will reduce the risk of diarrhoea faster than the performance of livestock and improve health (Krehbiel et al. 2003). In line with the current research, Ahmed et al. (2022) reported that adding 10 and 20 g of B probiotics to calf milk reduced the faeces score and diarrhoea. They also found that consumption of B by calves reduced the number of pathogenic microbes and increased the number of beneficial microbes, which prevented the occurrence of diarrhoea and increased faeces score (Mousa and Marwan 2019). Kowalski et al. (2009) and Agazzi et al. (2014) reported that the use of lactic acid bacteria in lactating animals reduced the incidence and frequency of diarrhoea and showed a slightly lower faeces score. Galvão et al. (2005) reported that calves receiving *S. cerevisiae* experienced fewer days of diarrhoea during pre‐suckling. Agazzi et al. (2014) and Kowalski et al. (2009) reported that the use of lactic acid bacteria in lactating cattle reduced the incidence and frequency of diarrhoea and showed a slightly lower faeces score. The results of studies show an improvement in faeces scores and a reduction in the frequency of diarrhoea in calves receiving yeast (Magalhães et al. 2008; Hill et al. 2009; Alugongo, Xiao, Wu, et al. 2017). However, some did not observe an effect on the faeces score (Lesmeister et al. 2004; Huuskonen and Pesonen 2015). In a study conducted on calves infected with *Salmonella enterica*, the yeast *S. cerevisiae* decreased the faeces score (Brewer et al. 2014; Harris et al. 2017). The addition of two percent DM of *S. cerevisiae* yeast has caused a significant decrease in faeces score and the number of days of diarrhoea (Magalhães et al. 2008). Aldana et al. (2009) and Cavini et al. (2015) reported that the addition of probiotics to the starter diet and milk of suckling calves resulted in improved faeces scores. In another report, they stated that the use of yeast (Heinrichs et al. 2003b) and *S. cerevisiae* yeast (Galvão et al. 2005; Fleige et al. 2007) in the milk of calves caused a numerical decrease in the faeces score and diarrhoea‐free days. It became calves. Also, many researchers' results show a significant reduction in health problems when calves receive probiotics (Abe et al. 1995; Abu‐Tarboush et al. 1996; ‏Timmerman et al. 2005).

Overall, the results of this research show that the effect of probiotics on the health of the digestive system of calves is minimal when the calves are healthy and well‐managed. By stimulating the non‐specific immune system, yeasts prevent the proliferation and growth of pathogenic bacteria in the digestive system and bind the oligosaccharide mannan present in the yeast wall to the intestinal epithelium and compete to remove the harmful bacteria attached to the epithelium; it improves livestock health index (Thomas et al. 2007; Izuddin et al. 2019). Also, *Bacillus* is capable of producing spores, which allows it to survive in adverse environmental conditions (such as high temperatures or low humidity). This feature allows the bacilli to easily reach and function in the intestines of calves. It can also boost beneficial bacteria in the gut and maintain a balance of gut flora. This balance can lead to a reduction in intestinal infections and improved overall health in calves (Ahmed et al. 2022).

Blood parameters due to the presence of homeostasis mechanisms and intense control by the nervous and glandular systems, blood parameters are not easily possible to change the metabolic factors of the blood and are affected by certain conditions such as malnutrition, infectious and parasitic diseases, insufficient nutrients in the diet compared to the minimum needs and conditions are placed like that (Alugongo, Xiao, Chung, et al. 2017). Before the calf becomes a ruminant, glucose is the main source of energy due to the limited use of volatile fatty acids in the underdeveloped rumen epithelium (Baldwin et al. 2004). An increase in energy consumption leads to an increase in glucose uptake (Magalhães et al. 2008), which is confirmed by Galvão et al. (2005) and Izuddin et al. (2019). Galvão et al. (2005) found that calves fed the yeast *S. cerevisiae* had higher glucose levels, both before and after suckling. However, in this study, calves also had a higher feed intake than the control group. Similarly, Izuddin et al. (2019) found that newly weaned lambs supplemented with L had significantly higher glucose levels compared to the control group. ‏Firooznia et al. (2019) reported that by adding 6 gr of yeast to the ration of dairy cows, the concentration of glucose increased significantly. Adding yeast to calves' diets also increased blood glucose concentration (Fouladgar et al. 2016; Noori et al. 2016; Le et al. 2016; Seifzadeh et al. 2017). This is probably due to the decrease in the molar share of acetic acid and the ratio of acetate to propionate and the increase in the molar share of propionic acid due to the better capacity of propionic acid bacteria to use lactate in rumen fluid of Holstein cows supplemented with yeast. Propionic acid is the main precursor of glucose in ruminants, and therefore, increasing the proportion of propionic acid in the rumen leads to providing glucose precursors and increasing glucose concentration. Probably, the lack of difference in the blood glucose concentration of the calves is due to the lack of difference in the fermentation level in the rumen, although they were numerically higher than the control treatment. When the fermentation level in the rumen increases, the starch in the rumen is fermented by the microorganisms in the rumen and turns into fatty acid, which is absorbed and metabolized by the rumen wall. However, the low level of fermentation in the rumen causes the starch to pass from the rumen to the intestine and is digested in the intestine and absorbed into the blood in the form of glucose (Hossein Abadi et al. 2021).

Blood urea nitrogen concentration is an indicator of rumen protein breakdown and post‐ruminal protein absorption (Nisbet et al. 1991; Miller‐Webster et al. 2002). Dolezal et al. (2005) reported in a study that the addition of *S. cerevisiae* caused a significant decrease in urea nitrogen levels. Norouzian et al. (2011) stated that consumption of *S. cerevisiae* yeast in lambs‐fed diets with 70% concentrate significantly reduced blood urea concentration 2 h after feeding. The reduction of blood urea nitrogen can be due to the high correlation between rumen and blood urea nitrogen (Offer 1990). Milewski and Sobiech (2009) observed that the concentration of urea nitrogen and plasma glucose was not affected by the daily feeding of *S. cerevisiae* yeast in the diet of dairy cows. Therefore, in the present experiment, due to the nutritional conditions of the calves used, as well as the provision of a suitable concentration of energy and protein and the similarity of the nutrients, no significant difference was observed in the concentration of urea, and this can show that the used yeasts affect. It did not affect microbial protein synthesis in calf rumen.

Regarding the cholesterol concentration, Cakiroglu et al. (2010) reported that the addition of a live yeast culture medium to the diet increased the cholesterol concentration in Jersey cows in early lactation. Piva et al. (1993) reported that blood cholesterol concentration was not affected by supplementing yeast culture medium. According to the results of this research, they stated that adding 6 g of *S. cerevisiae* yeast per day to the diet of Holstein dairy cows did not have a significant effect on the triglyceride concentration (‏Firooznia et al. 2019), although numerically, its decreased concentration was compared to the control group. Ayad et al. (2013) reported in a study that the addition of *S. cerevisiae* yeast significantly reduced the concentration of triglycerides. Consistent with our research, several researchers reported that the addition of yeast or yeast culture medium had no significant effect on blood triglyceride concentration (Milewski and Sobiech 2009; Cakiroglu et al. 2010; Ayad et al. 2012; Dehghan‐Banadaky et al. 2013). The lower concentration of triglyceride in animals fed with yeast is probably due to its negative correlation with the higher concentration of blood glucose (El‐Sherif and Assad 2001), which reduces the mobilization of fats from body tissues. The use of microbial additives also has a positive effect on energy balance by improving metabolic status, which reduces the concentration of serum lipids (Baiomy 2010). However, the concentration of triglyceride and cholesterol was not affected by the different experimental treatments, which indicates the balance of the metabolic status of the animals. In line with the results of the present study, Sallam et al. (2020) also reported that the use of yeast did not affect cholesterol concentration. Also, the contradiction in the obtained results can be related to the basic diet, the yeast strain used, the amount of yeast consumed and the physiological conditions of the animal under test (Williams et al. 1991; Patra 2012). The concentration of total protein, albumin and globulin indicates the level of protein metabolism in the animal. In this study, the concentration of total protein, albumin and globulin was not affected by the additives, which is consistent with the results of Galip (2006) and Soren et al. (2013). The lack of effect of the experimental treatments on the blood protein concentration indicates the proper nutritional status of the tested calves and the lack of use of amino acids and deamination to provide energy (Bruno et al. 2009).

Reference ranges for blood biochemical parameters in young calves indicate that the values observed in the present study were generally within the normal physiological limits reported in the literature. The primary objective of probiotic supplementation was not to increase or decrease these parameters, but rather to maintain normal metabolic status while improving growth performance and health. The lack of significant differences among treatments suggests that probiotic supplementation did not negatively affect the physiological condition of the calves and can be considered metabolically safe under the conditions of this experiment.

Several researchers stated that the use of yeast supplements in the diet improves skeletal growth (Galvão et al. 2005; Morrison et al. 2010), hip width, hip circumference, chest and abdomen environment (Lesmeister et al. 2004), hip height and hip height (Mir et al. 1994). Also, Ahmed et al. (2022) stated that adding yeast to the diet improved the hip width and the height of the withers of calves. Increased skeletal growth in calves receiving probiotics was probably the result of more available energy and nutrients for retention in the skeletons, which resulted from increased DMI. Morrison et al. (2010) stated that the increase in skeletal growth indices related to the increase in energy and protein consumption is due to the effect of probiotics on increasing the digestibility of food molecules. Because they have shown that probiotics improve feed intake and nutrient digestibility (Lesmeister et al. 2004).

Mohamadi Roodposhti et al. (2012) and Saremi et al. (2003) reported that consumption of *S. cerevisiae* yeast in nursing Holstein calves had no significant effect on the skeletal growth index. The addition of 1 and 2 gr of *S. cerevisiae* yeast to the milk and feed of calves until the age of 63 days did not have a significant effect on skeletal indices including withers height, body length, chest and hip‐width (Alugongo, Xiao, Chung, et al. 2017). Hill et al. (2009) did not observe any effect on wither height and breast girth by adding live yeast to the diet.

## 5 Conclusion

According to the results obtained in this research, the use of different probiotics improves DWG, DMI, FCR, overall performance, faeces score, digestibility of nutrients and skeletal index without negative effect on blood indices of calves. Among the different probiotics used in milk, *B. subtilis* had the best performance and improved the performance of calves. Therefore, probiotics can be used in the rations of suckling calves at the level of 5 gr.

## Author Contributions

**Mostafa Hosseinabadi**: conceptualization, methodology, writing – original draft. **Kamel Amozadeh Araee**: investigation, data curation, formal analysis. **Taghi Ghoorchi**: supervision, validation. **Ghasem Khadem**, **Ehsan Mirzadeh** and **Hamidreza Dosti**, resources, project administration.

## Funding

This study was financially supported by Vivan Tak ecsir Iranian Company (Mashhad, Iran) [Grant Number 586120].

## Ethics Statement

All animal handling and experimental procedures in this study were approved by the Animal Care and Use Committee of Gorgan University of Agricultural Sciences and Natural Resources (193408).

## Conflicts of Interest

The authors declare no conflicts of interest.

## Data Availability

Data will be made available on request.
